# Blood–brain barrier disruption and microglial activation during hypoxia and post-hypoxic recovery in aged mice

**DOI:** 10.1093/braincomms/fcae456

**Published:** 2024-12-17

**Authors:** Arjun Sapkota, Sebok K Halder, Richard Milner

**Affiliations:** San Diego Biomedical Research Institute, San Diego, CA 92121, USA; San Diego Biomedical Research Institute, San Diego, CA 92121, USA; San Diego Biomedical Research Institute, San Diego, CA 92121, USA

**Keywords:** blood–brain barrier integrity, aged, blood vessels, microglia, chronic mild hypoxia

## Abstract

Hypoxia triggers blood–brain barrier disruption and a strong microglial activation response around leaky cerebral blood vessels. These events are greatly amplified in aged mice which is translationally relevant because aged patients are far more likely to suffer hypoxic events from heart or lung disease, and because of the pathogenic role of blood–brain barrier breakdown in vascular dementia. Importantly, it is currently unclear if disrupted cerebral blood vessels spontaneously repair and if they do, whether surrounding microglia deactivates. In this study, we addressed these questions by exposing aged (20 months old) mice to chronic mild hypoxia (8% O_2_) for 7 days and then returned them to normoxic conditions for 7 or 14 days, before evaluating blood–brain barrier disruption and microglial activation at the different timepoints. Seven days chronic mild hypoxia triggered marked blood–brain barrier disruption, as measured by extravascular leak of fibrinogen and red blood cells, which led to enhanced microglial activation, as measured by Mac-1 and CD68 levels. Interestingly, while return to normoxia promoted spontaneous repair of damaged blood vessels, the surrounding microglia remained persistently activated and were slow to deactivate. Chronic mild hypoxia also triggered neuronal loss that resulted in irreversible cognitive decline as measured by the novel object recognition test. Taken together, these findings describe an important disconnect between vascular repair and microglial deactivation in aged mice, which likely contributes to prolonged neuroinflammation. As hypoxia occurs in many age-related conditions, our data have important implications for the pathogenic role of hypoxia in the induction and progression of vascular dementia.

## Teaser

Sapkota *et al.* show that hypoxia triggers vascular breakdown and microglial activation in the brain. While return to normoxia results in vascular repair, microglia remain activated for longer, demonstrating a disconnect between these two events. This results in neuronal death and cognitive decline.

## Graphical Abstract

**Graphical Abstract:**
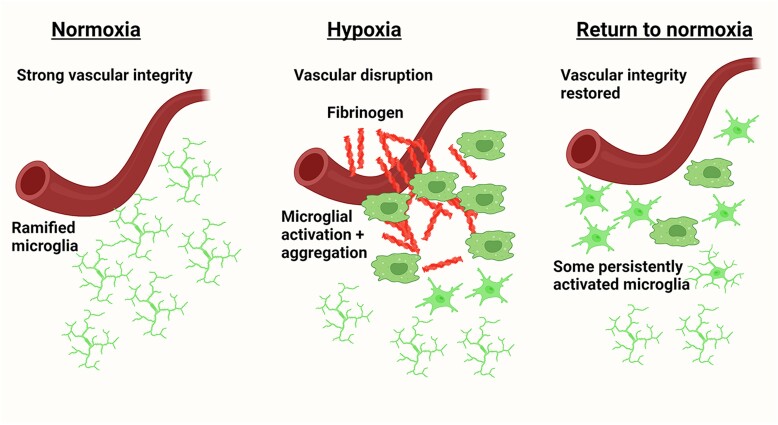

## Introduction

The blood–brain barrier (BBB) is an important anatomical and functional structure that precisely controls the homeostatic environment within the central nervous system (CNS).^1-3^ The BBB protects sensitive neural cells by precisely controlling the passage of substances into the CNS compartment. The molecular basis of the BBB relies on the adherents and tight junction protein complexes that formed between adjacent endothelial cells and the extracellular matrix components of the vascular basement membrane. Other CNS-resident cells such as astrocytes and pericytes play critical roles in enhancing BBB integrity.^4-6^ BBB disruption is a feature common to many neurological conditions, including ischaemic stroke, multiple sclerosis, meningitis and brain tumours.^6-8^ Emerging evidence suggests that BBB integrity also declines with increasing age and this BBB deterioration is thought to be an important contributor to the development and progression of vascular dementia.^9-12^

Hypoxia also triggers BBB disruption. Our laboratory and several others have shown that when mice are exposed to chronic mild hypoxia (CMH, 8% O_2_) for 7 days, this triggers a marked cerebral angiogenic response that correlates with transient BBB disruption. In a series of recent studies, we have shown that this hypoxia-induced BBB disruption is strongly associated with microglial activation and aggregation around the disrupted cerebral blood vessels.^13-15^ Interestingly, both BBB disruption and microglial activation are greatly enhanced in aged mice, as shown by a 5–10-fold greater density of vascular leaks. This is highly relevant from a translational perspective because aged patients are far more likely to experience hypoxia due to greater incidence heart and lung disease in the elderly, and because of the pathogenic role of BBB breakdown in vascular dementia. In contrast to our own findings of a close relationship temporal and spatial relationship between BBB disruption and microglial activation, a recent MRI study of cerebral small vessel disease (SVD) in patients, found BBB disruption and microglial activation to be spatially unrelated, suggesting that these two events are independent contributors to SVD.^16^

As our results show that hypoxia-induced BBB breakdown and microglial activation both greatly increase with age, this supports the idea that with increasing age, cerebral blood vessels are more susceptible to disruption in the face of hypoxic insult, resulting in greater levels of microglial activation.^17^ However, a clinically important question that has not been addressed to date, is what happens to cerebral blood vessels that have been disrupted. Do they remain permanently leaky, or do they spontaneously repair over time, and how does this correlate with changes in microglial activation state? To answer this question, we designed a simple study in which aged (20 months old) mice were exposed to CMH for 7 days, sufficient time to induce marked BBB breakdown and microglial activation in these elderly subjects. We then analysed levels of BBB breakdown (using extravascular leak of fibrinogen and red blood cells), and microglial activation (using the microglial activation markers Mac-1 and CD68), either immediately following 7 days hypoxia, or 7 or 14 days after return of the mice to normoxic conditions. This approach was designed to model what happens in an aged patient who experiences hypoxia due to cardiac or pulmonary insufficiency,^18^ and then receives medical treatment to overcome the hypoxia. The goal of this study was to address three specific questions highly relevant to the aged brain: (i) what is the spatial and temporal association between BBB disruption and microglial activation during the period of hypoxic insult and in the normoxic recovery period that follows: (ii) does the disrupted BBB repair over time and (iii) if vascular leaks are repaired, do activated microglia in the vicinity of vascular leaks revert to their pre-hypoxic resting state?

## Materials and methods

### Animals

The studies described were reviewed and approved by the Institutional Animal Care and Use Committee at San Diego Biomedical Research Institute (SDBRI). Aged 20-months-old female C57BL6/J mice were obtained from the NIH National Institute on Aging rodent colony and were maintained under pathogen-free conditions in the closed breeding colony of SDBRI.

### Chronic hypoxia model

Aged 20-months-old female C57BL6/J mice were housed four to a cage, and placed into a hypoxic chamber (Biospherix, Redfield, NY, USA) maintained at 8% O_2_ (equivalent to an altitude of ∼24 000 feet elevation). Seven days later, they were either euthanized immediately, or switched back to normoxic conditions for 7 or 14 days before being euthanized. Another group of control mice were kept in the same room under similar conditions except that they were kept at ambient sea-level oxygen levels (normoxia, ∼21% O_2_ at sea-level) for the duration of the experiment. Every few days, the chambers were briefly opened for cage cleaning and food and water replacement as needed. We used 20-months-old mice because in mice, aging is defined as the period between 18 and 24 months, when almost all biomarkers of senescence can be detected in all animals.

### Novel object recognition test

This test is based on the tendency of mice to explore a novel object more than a familiar one, and consists of three stages: habituation, training and testing.^19^ During the habituation stage, mice were allowed to habituate to the test cage for 10 min. Shortly afterwards, in the training stage, they were allowed to interact with two identical objects for 5 min then returned to their home cage for a 1-h retention period. Finally, in the testing stage, one of the familiar objects was replaced with a novel one and the time spent investigating each object was recorded. The discrimination index was then calculated, and data analysed with ANOVA. All behavioural studies were performed on aged (20 months old) female C57BL6/J mice using *n* = 6–14 mice per group.

### Immunohistochemistry and antibodies

Immunohistochemistry was performed on 10 µm frozen sections of cold phosphate buffer saline perfused tissues as described previously.^20,21^ Briefly, at the termination of the study, mice were subject to deep anaesthesia using a cocktail of ketamine and xylazine, and transcardially perfused. Rat monoclonal antibodies from BD Pharmingen (La Jolla, CA, USA) reactive for the following antigens were used in this study: CD31 (clone MEC13.3; 1:300), MECA-32 (1: 100), Mac-1 (clone M1/70; 1:50) and CD68 (clone FA-11; 1:2000). Rat anti-TER-119 was obtained from R&D Systems (clone FA-11; 1:500). The hamster anti-CD31 (clone 2H8; 1:500) and rabbit anti-NeuN (clone EPR12763; 1:4000) monoclonals were obtained from Abcam (Cambridge, MA, USA). The rabbit antibody reactive for fibrinogen (1:2000) was obtained from Millipore (Temecula, CA, USA). Sheep anti-fibrinogen (1:3000) was obtained from Bio-Rad. Secondary antibodies used (all at 1:500) include Cy3-conjugated anti-rabbit, anti-rat, anti-mouse, anti-sheep and Cy5-conjugated anti-rabbit from Jackson Immunoresearch, (West Grove, PA, USA) and Alexa Fluor 488-conjugated anti-rat, anti-hamster and anti-rabbit from Invitrogen (Carlsbad, CA, USA). Fluoromyelin red (1:50) was obtained from Invitrogen.

### Image analysis

Images were taken using an Axioskop2 plus microscope (Carl Zeiss, Dublin, CA, USA) equipped with an Infinity 3S camera (Lumenera, Ottawa, ON, Canada) and Infinity Analyse imaging software (Lumenera). For each antigen in all analyses, images of at least three randomly selected areas were taken at 5×, 10× or 20× magnification per tissue section and three sections per brain analysed to calculate the mean for each animal (*n* = 6 mice per group). The number of vascular leaks, haemorrhages or MECA-32 + vessels per field of view (FOV) were quantified by capturing images and performing manual counts of the number of vessels showing extravascular leaked fibrinogen, TER-119 or MECA-32. The number of CD68^+^ cells per FOV was quantified by performing manual counts. Total Mac-1 fluorescent signal per FOV was measured and analysed using NIH Image J software.

### Statistical analysis

Each experiment was performed with six different animals per condition, and the results expressed as the mean ± standard error of the mean (SEM). Statistical significance was assessed using one-way ANOVA followed by Tukey’s multiple comparison *post hoc* test, in which *P* < 0.05 was defined as statistically significant.

## Results

### In aged mice, CMH triggers BBB breakdown and haemorrhage that is strongly associated with microglial activation

**Figure 1:**
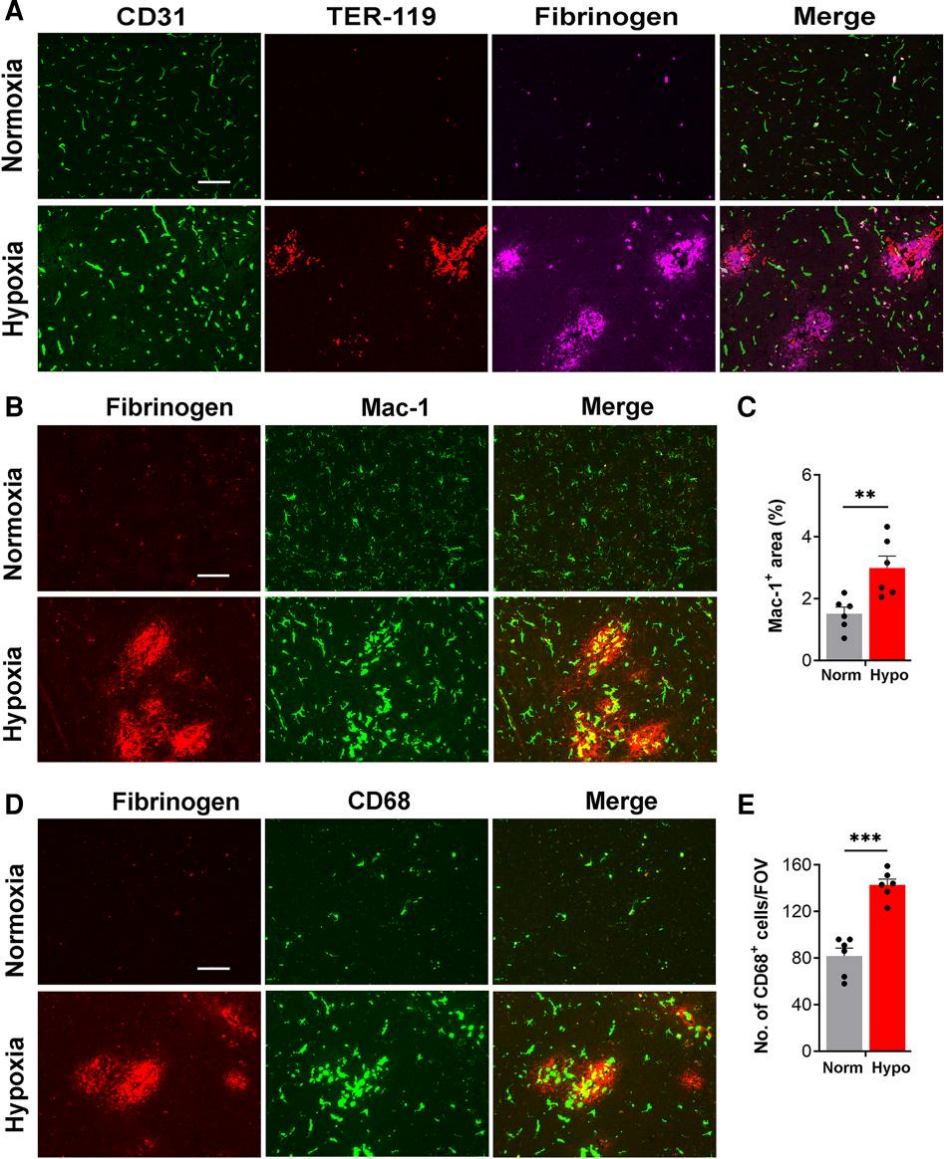
**Hypoxia-induced BBB disruption is strongly associated with microglial activation.** Frozen brain sections taken from aged (20 months) mice exposed to normoxia (Nor) or 7 days hypoxia (8% O_2_) were stained for the endothelial marker CD31 (AlexaFluor-488), the RBC marker TER-119 (Cy-3) and fibrinogen (Cy-5) (**A**), fibrinogen (Cy-3) and Mac-1 (AlexaFluor-488) (**B**), or fibrinogen (Cy-3) and CD68 (AlexaFluor-488) (**D**). Images were captured in the mid-brain. Scale bars = 100 μm. Quantification of Mac-1^+^ area (**C**) or number of CD68^+^ cells/FOV (**E**) following Nor or 7 days hypoxia. Results are expressed as the mean ± SEM (*n* = 6 mice/group). **P < 0.01, ***P < 0.001. One-way ANOVA followed by Tukey’s multiple comparison *post hoc* test. Note that hypoxia triggered both extravascular leak of fibrinogen and haemorrhage, as indicated by the RBC marker TER-119. BBB disruption was strongly associated with microglial activation as shown by aggregation and morphological transformation of Mac-1 and CD68^+^ cells around the fibrinogen^+^ leaks.

Aged (20 months old) mice were exposed to 7 days CMH before tissues were harvested. Frozen brain sections were examined by triple-immunofluorescence (IF) to detect blood vessels (CD31), extravascular leak (fibrinogen) and haemorrhage [using the red blood cell (RBC) marker TER-119^22^]. As shown in Fig. 1A, while no vascular leak was seen in normoxic mice, CMH triggered BBB disruption in several blood vessels, and this was often accompanied by haemorrhage. Dual-IF with fibrinogen and the microglial marker Mac-1 revealed that disrupted blood vessels were strongly associated with accumulation and aggregation of microglia displaying the activated morphological phenotype (large cell body and short processes), resulting in a much stronger Mac-1 signal (Fig. 1B and C). Staining with CD68, a lysosomal marker of microglial priming,^23^ confirmed these findings, showing an increased density and size of CD68^+^ cells in the region of vascular leak (Fig. 1D and E).

### Hypoxia-induced BBB disruption is associated with neuronal loss

**Figure 2:**
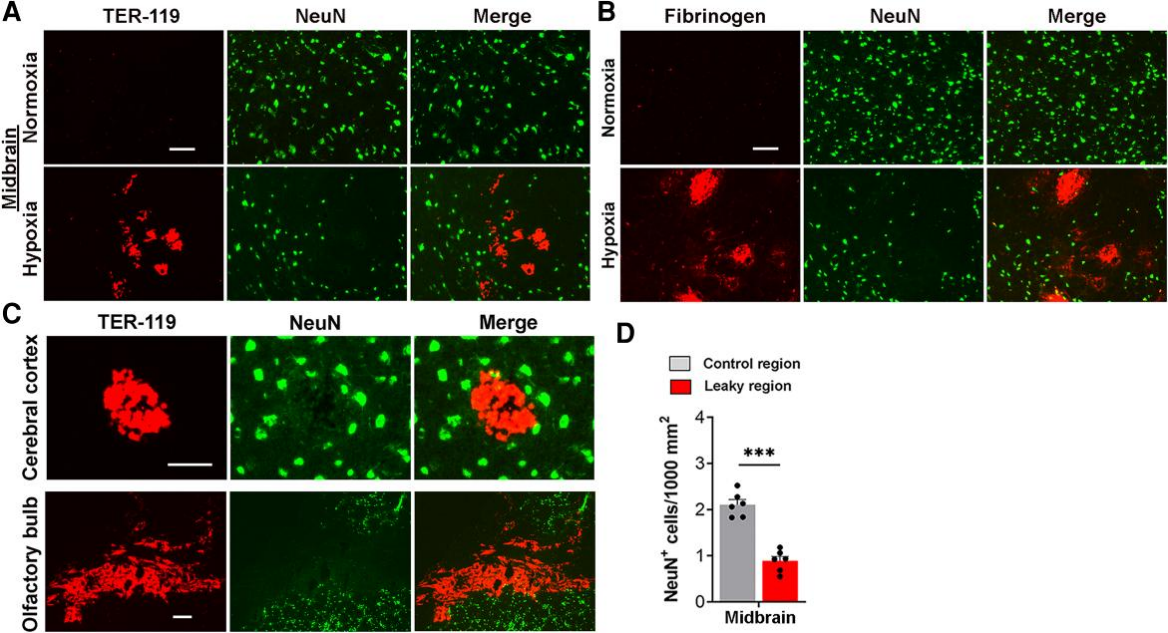
**Hypoxia-induced BBB disruption is associated with neuronal loss.** Frozen brain sections taken from aged (20 months) mice exposed to Nor or 7 days hypoxia (8% O_2_) were stained for TER-119 (Cy-3) and NeuN (AlexaFluor-488) (**A**, mid-brain), fibrinogen (Cy-3), and NeuN (AlexaFluor-488) (**B**, mid-brain), or TER-119 (Cy-3) and NeuN (AlexaFluor-488) (**C**, cerebral cortex and olfactory bulb). Scale bars = 100 μm (**A**, **B**), 50 μm (top row of **C**), or 200 μm (lower row of **C**). (**D**) Quantification of neuronal density (NeuN^+^ cells/1000 μm^2^) in the mid-brain after 7 days hypoxia. Results are expressed as the mean ± SEM (*n* = 6 mice/group). ***P < 0.001. One-way ANOVA followed by Tukey’s multiple comparison *post hoc* test. Note that this revealed a strong relationship between extravascular leak of fibrinogen or red blood cells, and reduced density of neurones in all three brain regions examined.

To determine if hypoxia-induced BBB disruption impacts neuronal viability in aged mice, we next performed dual-IF with the neuronal marker NeuN in conjunction with the vascular disruption markers, TER-119 and fibrinogen. This revealed a strong relationship between extravascular leak of fibrinogen or red blood cells, and reduced density of neurones. This was particularly striking in the mid-brain (Fig. 2A and B) but was also evident in the cerebral cortex and olfactory bulb (Fig. 2C). In all regions examined, it was noticeable that areas showing BBB disruption were devoid of neuronal cells in the immediate vicinity. Quantification of this in the mid-brain showed that neuronal density was significantly reduced in areas containing vascular leaks (Fig. 2D).

### Return to normoxic conditions stimulates repair of disrupted blood vessels and myelin

**Figure 3:**
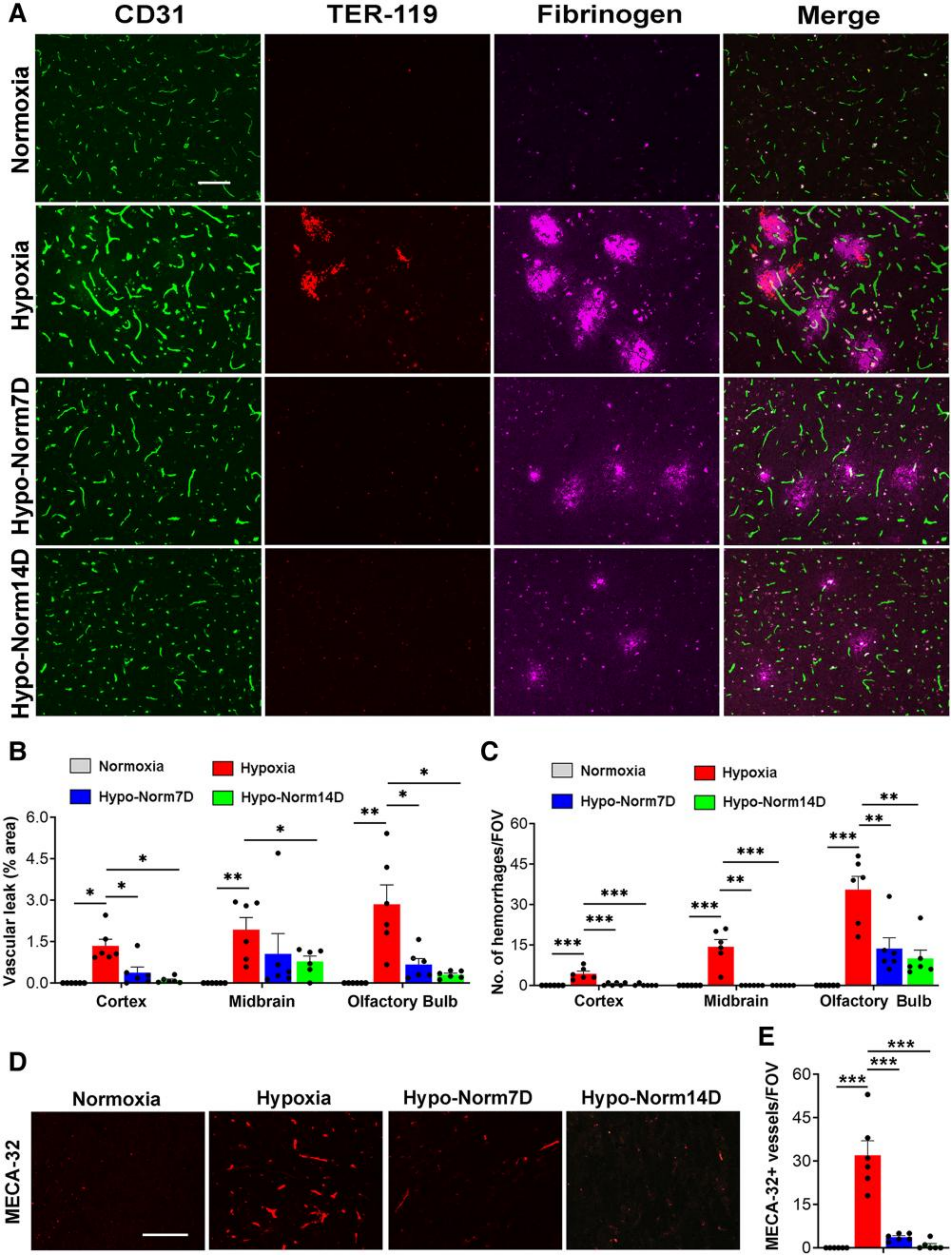
**Hypoxia-induced BBB disruptions spontaneously repair under normoxic conditions.** Frozen brain sections taken from aged (20 months) mice exposed to Nor, 7 days hypoxia (8% O_2_), or 7 days hypoxia followed by an additional 7- or 14-days at normoxic conditions (Hypo-Norm7D and Hypo-Norm14D, respectively), were stained for (**A**) CD31 (AlexaFluor-488), TER-119 (Cy-3) and fibrinogen (Cy-5) or (**D**) MECA-32. Images were captured in the mid-brain. Scale bars = 100 μm. (**B**, **C** and **E**) Quantification of the area of fibrinogen^+^ vascular leak (**B**), number of TER-119^+^ haemorrhages (**C**), or density of MECA-32 + vessels (**E**) following the different treatments. Results are expressed as the mean ± SEM (*n* = 6 mice/group). *P < 0.05, **P < 0.01, ***P < 0.001. One-way ANOVA followed by Tukey’s multiple comparison *post hoc* test. Note that 7 days hypoxia triggered extravascular leak of fibrinogen and haemorrhage as well as high endothelial MECA-32 induction but return to normoxic conditions resulted in significant disappearance of all these markers.

**Figure 4:**
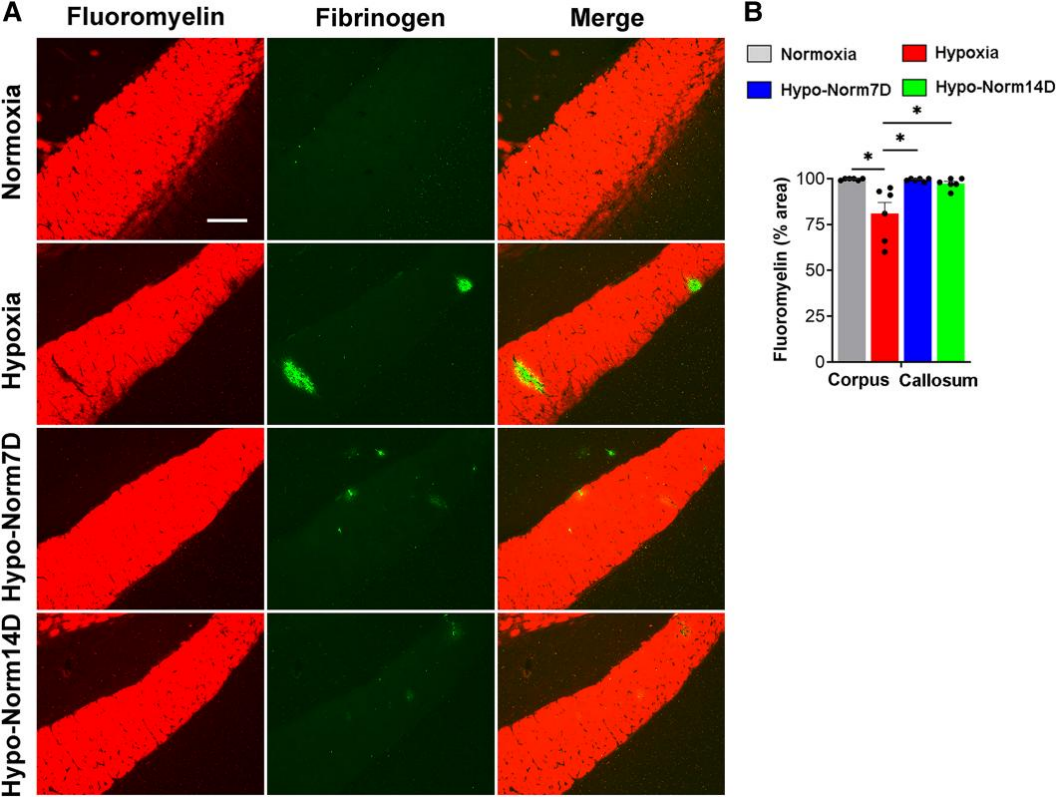
**Hypoxia-induced demyelinated lesions spontaneously repair after return to normoxic conditions.** (**A**) Frozen brain sections taken from aged (20 months) mice exposed to Nor, 7 days hypoxia (8% O_2_), or 7 days hypoxia followed by an additional 7- or 14-days at normoxic conditions (Hypo-Norm7D and Hypo-Norm14D, respectively), were stained with fluoromyelin red and fibrinogen (AlexaFluor-488). Images were captured in the corpus callosum. Scale bar = 200 μm. (**B**) Quantification of the fluoromyelin^+^ area in the different experimental groups. Results are expressed as the mean ± SEM (*n* = 6 mice/group). *P < 0.05. One-way ANOVA followed by Tukey’s multiple comparison *post hoc* test. Note that 7 days hypoxia triggered extravascular leak of fibrinogen that strongly associated with loss of myelin but return to normoxic conditions resulted in marked reduction in fibrinogen staining and recovery of myelin integrity.

To determine if hypoxia-induced disrupted blood vessels are capable of spontaneous repair, aged mice were exposed to CMH for 7 days and the extent of BBB breakdown was evaluated, immediately after 7 days hypoxic exposure, or after an additional 7- or 14-days following return to normoxic conditions. As shown in Fig. 3A (mid-brain), mice analysed immediately after 7 days hypoxia showed multiple large fibrinogen^+^ regions, many of which also showed evidence of TER-119^+^ RBC deposition, indicating extensive BBB disruption. In sharp contrast, mice that were exposed to hypoxia but then returned to normoxic conditions for 7 or 14 days, showed a total absence of TER-119 staining. Some feint fibrinogen staining was still detectable, but the overall signal intensity was much weaker than immediately after hypoxia, implying that the feint fibrinogen staining may be a remnant of previous BBB damage yet to be adequately cleared. Analyses of the cerebral cortex and olfactory bulb revealed similar findings. Quantification in all regions examined demonstrated that mice returned to normoxic conditions for 7 or 14 days showed greatly reduced vascular leak, implying that hypoxia-induced BBB disruptions are actively repaired following return to normoxic conditions. In addition, the evaluation of MECA-32, a well-established marker of leaky BBB,^24,25^ confirmed that the density of MECA-32^+^ vessels increased sharply after 7 days hypoxia, but after 7 or 14 days return to normoxic conditions, MECA-32 expression virtually disappeared (Fig. 3D and E). Given the importance of myelin for learning and cognitive function, we next examined how hypoxia-induced vascular leak impacts myelin integrity in the well-defined myelinated tract, the corpus callosum. Dual-IF with fibrinogen and fluoromyelin revealed that in mice exposed to hypoxia, extravascular leak of fibrinogen was strongly associated with loss of myelin integrity (Fig. 4A). Interestingly however, in the brains of mice returned to normoxic conditions for 7 or 14 days, the disappearance of extravascular fibrinogen leak coincided with repair of myelin integrity, such that demyelinated lesions were undetectable in these mice following return to normoxic conditions (Fig. 4A and B).

### Microglia are slow to deactivate upon return to normoxic conditions

**Figure 5:**
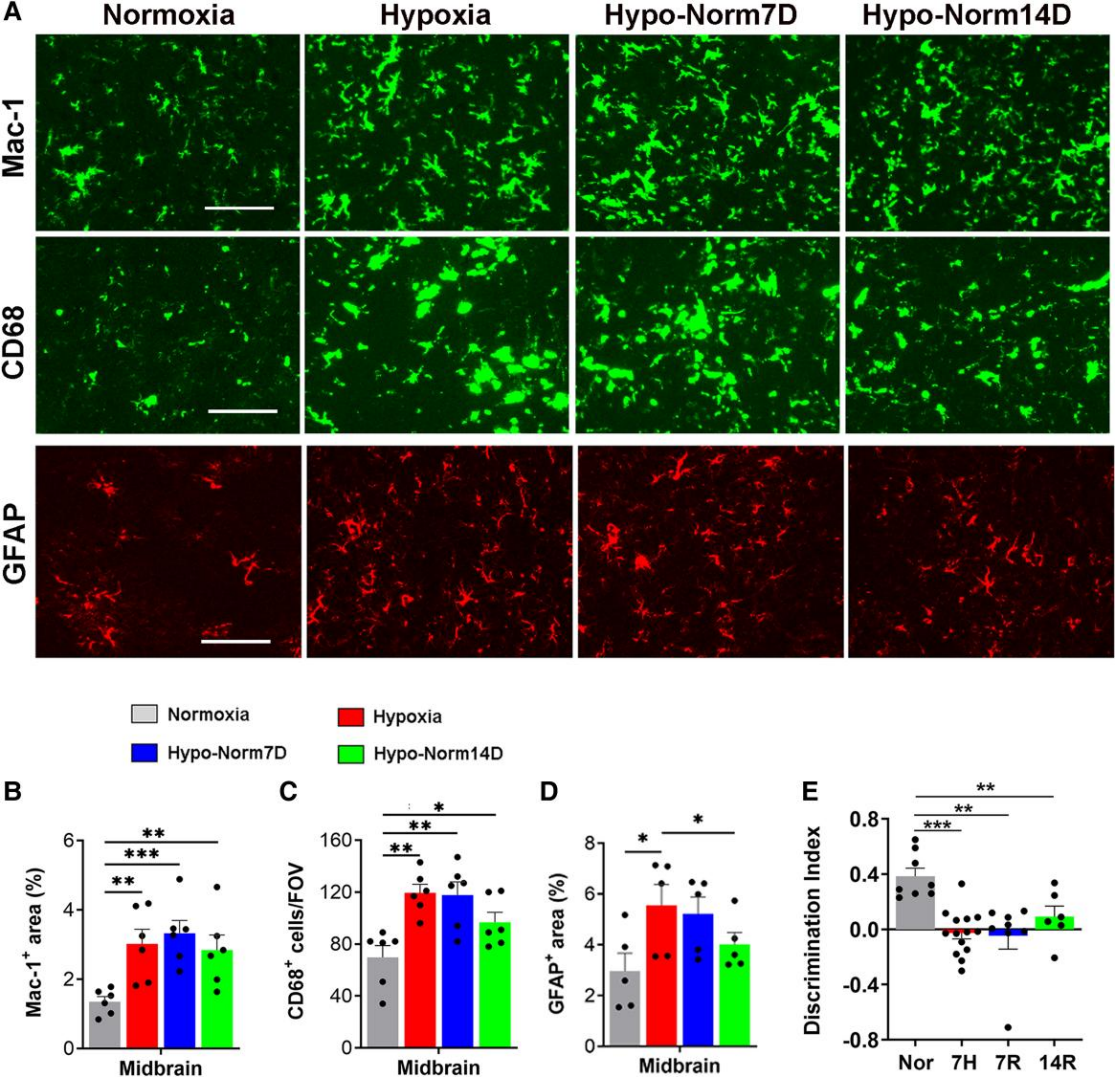
**Hypoxia-induced microglial activation and cognitive function are slow to reverse after return to normoxic conditions.** (**A**) Frozen brain sections taken from aged (20 months) mice exposed to Nor, 7 days hypoxia (8% O_2_), or 7 days hypoxia followed by an additional 7- or 14-days at normoxic conditions (Hypo-Norm7D and Hypo-Norm14D, respectively), were stained for Mac-1, CD68 or GFAP. Images were captured in the mid-brain. Scale bar = 100 μm. (**B–D**) Quantification of Mac-1^+^ area (**B**) number of CD68^+^ cells/FOV (**C**) or GFAP area (**D**) following the different treatments. Results are expressed as the mean ± SEM (*n* = 6 mice/group). *P < 0.05, **P < 0.01, ***P < 0.001. Note that 7 days hypoxia triggered strong microglial and astrocyte activation, but that 7- or 14-days after return to normoxic conditions, microglia, but not astrocytes, remained significantly activated. (**E**) NOR test on aged mice exposed to Nor, 7 days hypoxia (7H), or 7 days hypoxia followed by 7 (7R) or 14 (14R) days of recovery under normoxic conditions. Results are expressed as the mean ± SD (*n* = 6–14 mice/group). **P < 0.01, ***P < 0.001. One-way ANOVA followed by Tukey’s multiple comparison *post hoc* test. Note that 7 days hypoxia significantly reduced the discrimination index, and that cognitive decline remained 14 days after return to Nor.

When we examined how return to normoxic conditions affects microglial activation, we were surprised to see that they were slow to deactivate. Specifically, 14 days after return to normoxic conditions, Mac-1 expression remained elevated compared with the pre-hypoxic level and was largely equivalent to the level immediately after 7 days hypoxia (Fig. 5A and B). In support of this, microglia in the brains of these mice continued to display the highly activated morphology (large cell body with short process extensions). Consistent with this, in mice returned to normoxic conditions, the density of CD68^+^ microglia remained significantly higher than control pre-hypoxic levels, even as late as 14 days after return to normoxia (Fig. 5A and C). These results demonstrate a disconnect between BBB breakdown and microglial activation in the aged brain, in that while return to normoxia for 7 or 14 days promotes the repair of damaged cerebral blood vessels, microglia remain activated for an extended period. As astrocytes also react to hypoxia and other insults, we next evaluated how hypoxia and subsequent return to normoxia influences astrocyte activation. As shown in Fig. 5A (lower panel), 7 days of hypoxia triggered a marked increase in glial fibrillary acidic protein (GFAP) signal but this gradually reduced following return to normoxic conditions, such that 14 days after return to normoxic conditions, the GFAP signal was significantly lower than that following 7 days hypoxia and not significantly any different from that under normoxic conditions (Fig. 5A and D).

### CMH triggers cognitive decline in aged mice that is not reversed upon return to normoxic conditions

To evaluate the impact of CMH and return to nornoxic conditions on cognitive function, we employed the novel object recognition (NOR) test, which tests the mouse’s ability to recognize and remember a familiar object previously encountered.^19^ Because rodents have a preference of novelty, one that remembers the familiar object will tend to spend more time exploring the novel object.^26^ Using this behavioural test, we found that aged mice maintained under normoxic conditions could easily discriminate between familiar and novel objects, resulting in a relatively high discrimination index (Fig. 5E). By contrast, aged mice exposed to CMH for 7 days lost the ability to discriminate between familiar and novel objects. More importantly, and highly relevant to the goals of this study, aged mice returned to normoxia for 7 or 14 days, showed no signs of recovery in this behavioural test. This suggests that the neuronal damage that occurred during the 7-day hypoxic period was not reversed by return to normoxic conditions.

## Discussion

In previous studies, we demonstrated that CMH (8% O_2_) disrupts BBB integrity, resulting in leakage of blood proteins (including fibrinogen) into the brain parenchyma, which in turn promotes a very strong microglial activation response specifically around the extravascular leak.^14^ Importantly, both BBB disruption (as measured by extravascular fibrinogen leak) and microglial activation are greatly amplified in the brains of aged mice, such that CMH triggers 5–10-fold the number of BBB disruptions in aged versus young mice.^17^ Considering that hypoxia-induced BBB disruption is much more severe in aged mice, and the pathogenic potential of hypoxic insult in triggering neurodegeneration in aged patients, the goal of this study was to model these events in aged mice to determine what happens to cerebrovascular leaks. With this in mind, we set out to address the specific questions: do blood vessels repair when normoxic conditions are restored and do microglia revert to their unactivated phenotype? Our main findings were as follows: (i) CMH triggers BBB disruption that is strongly associated with adjacent microglial activation and aggregation, (ii) return to normoxia promotes spontaneous repair of disrupted blood vessels, but activated microglia are slow to deactivate and (iii) hypoxia triggers neuronal loss in parallel with a permanent decline in cognitive function.

### BBB integrity and microglial activation

Using the CMH model, we have demonstrated that mild hypoxic insult disrupts BBB integrity, which in turn stimulates microglial activation.^14^ Importantly, both these events are greatly amplified in the brains of aged mice.^17^ Of note, even under normoxic control conditions, microglia in aged mice are much more activated than their younger counterparts, and CMH triggers 5–10-fold the number of BBB disruptions in aged versus young mice.^17^ What is not yet clear is whether hypoxia-damaged blood vessels spontaneously repair over time, or whether microglia revert to their pre-hypoxic level of activation. The studies we present here were largely motivated by recent observations in SVD patients that revealed evidence of ongoing BBB disruption and microglial activation, but surprisingly, these two events were found to be spatially distinct processes, prompting the conclusion that these two pathological events are independent contributors to SVD.^16^ These findings are at odds with our own demonstration of an extremely tight spatial and temporal relationship between BBB disruption and microglial activation in the murine CMH model.^14,17,27^ Several factors might account for the perceived difference in our findings. First, the time course of the two types of study is very different. While our mouse studies are typically performed over 2–3 weeks, the data obtained in patients likely represent events that have occurred over a much longer time course. Second, our mouse studies use a very defined and strong hypoxic stimulus, while the patient data likely represent a wider range of hypoxic stimuli of differing severity. Importantly, the findings from our current study confirm our previous findings that in response to hypoxia, BBB disruption and microglial activation strongly correlate. Interestingly however, in the normoxic recovery period, we noted a disconnect between BBB disruption and microglial activation such that after 14 days normoxic recovery, very little signs of BBB disruption were evident, but notably, microglia remained significantly elevated. These findings demonstrate that while vascular repair proceeds relatively quickly, microglia remain activated for extended periods of time and are slow to revert to their pre-hypoxic activation state. The data we present here suggest an alternative explanation for the findings from human SVD patients. Namely, any snapshot in time could give the potentially misleading impression that BBB disruption and microglial activation are unrelated phenomena, because newly emerging vascular leaks may not have yet triggered a strong microglial response, while equally, resolving vascular leaks might show no vascular leak but still display strong adjacent residual microglial activation. It is important to consider that although our studies demonstrate that hypoxia-damaged blood vessels can initiate self-repair following return to normoxic conditions, it remains possible that these vessels have not been fully repaired back to the pre-hypoxic normal state. This is because the markers we used for detecting vascular disruption (fibrinogen and RBCs) are all relatively large; thus, it is possible that the BBB in these mice is still relatively permeable to other smaller molecular weight markers.

### Implications of repeated hypoxic insults to the aged brain

Our recent studies have clearly demonstrated that hypoxic insults trigger BBB disruption, and importantly, that cerebral blood vessels in the aged brain are far more susceptible to this breakdown.^14,17^ Our new findings add to this concept in three ways. First, they make the important point that disrupted blood vessels are capable of spontaneous repair. Second, despite this vascular repair, the microglia can remain chronically activated, thereby elevating the neuroinflammatory state, which could predispose to further pathological demise. Third, hypoxia-induced BBB disruption results in irreversible neuronal loss in multiple areas of the brain, culminating in significant cognitive decline. Considering that hypoxia occurs in many conditions in aged patients, including asthma, sleep apnoea, chronic obstructive pulmonary disease and heart failure, many of which increase the risk of cognitive decline,^18,28-30^ our findings suggest that repeated hypoxic episodes may significantly contribute to the progression of vascular dementia. This knowledge raises the question: how can we enhance BBB integrity in aged patients to prevent hypoxic vascular disruption and microglial activation, and thereby forestall the inevitable age-related neurodegeneration and cognitive decline? Recent studies have yielded several cellular and molecular targets that may prove fruitful. First, as the mural cells, pericytes play an important role in promoting BBB integrity,^4^ taken with recent evidence that pericyte number declines with age,^31,32^ one approach would be to promote pericyte density or maintenance at the BBB. Second, considering the critical role of endothelial tight junction proteins at the BBB,^33,34^ approaches to increase their expression or stability may reinforce BBB integrity. Third, our recent studies suggest that overly activated microglia in the brain of aged mice are less effective at vasculo-protection, but that attenuation of microglial activation restores this function and reduces hypoxic BBB disruption.^17^ Further elucidation of these pathways will clarify their future clinical potential in the treatment of vascular dementia and other neurological conditions.

## Conclusion

While it is well established that hypoxia triggers BBB disruption that is strongly associated with microglial activation and aggregation around the leaky blood vessels, it is currently unknown if disrupted cerebral blood vessels spontaneously repair and if they do, whether the surrounding activated microglia deactivate. Here, we addressed these questions by exposing aged mice to CMH for 7 days and then returned them to normoxic conditions for 7 or 14 days, before evaluating BBB disruption and microglial activation at the different timepoints. This showed that 7 days CMH triggered marked BBB disruption which was closely associated with microglial activation. Interestingly, while return to normoxia promoted spontaneous repair of damaged blood vessels, the leak-associated microglia remained persistently activated and were slow to deactivate. CMH also triggered neuronal loss in several brain regions that resulted in irreversible cognitive decline. These findings describe an important disconnect between vascular repair and microglial deactivation in aged mice, which may contribute to prolonged neuroinflammation. As hypoxia occurs in many age-related conditions, our data have important implications for the pathogenic role of hypoxia in the induction and progression of vascular dementia.

## Data Availability

The datasets used and/or analysed during the current study are available from the corresponding author upon reasonable request.
